# Oral Doxycycline Reduces the Total Number of Intraocular Bevacizumab Injections Needed to Control Neovascular Age-related Macular Degeneration

**Published:** 2017

**Authors:** Ahmad MIRSHAHI, Pourya AZIMI, Ali ABDOLAHI, Romina MIRSHAHI, Mahnaz ABDOLLAHIAN

**Affiliations:** 1 Eye Research Center, Farabi Eye Hospital, Tehran University of Medical Science, Tehran, Iran; 2 Student Scientific Research Center, Tehran University of Medical Sciences, Tehran, Iran

**Keywords:** Macular Degeneration, Bevacizumab, Doxycycline, Neovascularization

## Abstract

Tetracyclines, especially doxycycline, play a role in the regulation of inflammation, immunomodulation, cell proliferation, and angiogenesis. Treatment of corneal angiogenesis or choroidal neovascularization with tetracyclines has been shown to be effective in animal models. The aim of this study was to evaluate the efficacy of oral doxycycline in reducing the total number of intraocular injections needed for controlling neovascular age-related macular degeneration in human patients. In this interventional case series, 28 random consecutive patients with neovascular age-related macular degeneration from Farabi Eye Hospital, Tehran, Iran were treated for 4 months with 200 mg doxycycline once a day after the first intravitreal bevacizumab injection in addition to standard therapy in agreement with as-needed regimen. After 12 months of follow-up, total number of injections, foveal thickness and visual acuity were compared to those at baseline and of similar studies. Similar to standard treatment, co-treatment with doxycycline was able to control active disease (intraretinal or subretinal fluid or leakage, new-onset of macular hemorrhage, and reduction of visual acuity more than 5 letters based on Early Treatment Diabetic Retinopathy Study [ETDRS] charts) yet with fewer injections (for current study and standard treatment, respectively 3.14 vs. 5.92, P < 0.001). Furthermore, while better control of the foveal thickness was achieved (P < 0.001), vision improvement was similar to that achieved with standard therapy (P > 0.05). If confirmed in larger studies, the findings of this interventional case series could provide a strategy to control neovascular age-related macular degeneration with fewer intraocular bevacizumab injections by co-administering a well-known oral agent—doxycycline.

## INTRODUCTION

In developed countries, age-related macular degeneration (AMD) is the foremost reason of legal blindness among individuals over 50 years old. In AMD, central vision impairment is engendered due to a degenerative process of the macula. Since AMD is a multifactorial spectrum of macular degeneration, at the point of imbalance between inhibiting and promoting neovascularization factors, neovascular AMD (nAMD) may occur. Inhibition of vascular endothelial growth factor (VEGF) by numerous intravitreal injections has been the basis of nAMD treatment over the past 10 years [1]. Anti-VEGF compounds, such as pegaptanib, bevacizumab, ranibizumab, and aflibercept, are commonly used to treat nAMD. However, in spite of treatment strategies such as treat-and-extend or as-needed (pro re nata [PRN]) regimens, management of choroidal neovascularization (CNV) is still a dilemma [2]. Alongside the results of clinical studies, outcomes of everyday practice show that a treatment regimen with frequent injections may be less favorable and even pose an enormous burden on health care systems [3]. To reduce the need for multiple intraocular injections, some authors have suggested co-treatments should be considered for patients [1, 4].

In addition to their well-known bacteriostatic properties, tetracyclines, especially doxycycline and minocycline, regulate inflammation, immunomodulation, cell proliferation, and angiogenesis. In particular, the anti-angiogenic properties of doxycycline—which have been recognized since the 1990s—have been shown to play a role in the treatment of pathologic angiogenesis [5]. Such properties have encouraged us to conduct an interventional case series as a pilot stage for a randomized control trial (RCT) to improve management of CNV by administration of doxycycline. To our knowledge, this is the first case series of its type in human subjects. The findings of this case series could provide a strategy to reduce the total number of intraocular bevacizumab injections and related problems by co-administering a well-known oral agent—doxycycline.

## MATERIALS AND METHODS

A total of 28 random consecutive patients from Farabi Eye Hospital, Tehran, Iran met the inclusion criteria (Table 1) and were enrolled in this interventional case series. The study was conducted between October 2014 and March 2015. Each patient signed an informed consent form that was provided by our ethics committee in agreement with the Declaration of Helsinki.

According to our conventional treatment protocol for active lesions of nAMD, patients were treated with 1.25mg bevacizumab (Avastin®, Genentech, South San Francisco, CA, USA) by a single intravitreal injection in a volume of 0.05 cc under topical anesthesia in the operation room. To check for any sign of disease activation or indication for re-treatment, patients were evaluated monthly for 6 months and thereafter every 3 months for the subsequent 6 months. In each session, the following examinations were performed: fundus examination, best-corrected visual acuity (BCVA), anterior chamber examination, and optical coherence tomography (OCT; Spectralis®, Heidelberg Engineering, Heidelberg, Germany). Fluorescein angiography (FA; HRA 2, Heidelberg Engineering) was requested in the first visit and in special cases during follow-up. In case of suspicion of retinal angiomatous proliferation or polypoidal choroidal vasculopathy, indocyanine green (ICG) imaging was performed. Re-treatment indications are listed in Table 1. The re-treatment dose of intravitreal bevacizumab was equal to the initial dose. In the case of scar formation or lack of re-treatment indication, lesions were considered as inactive and patients were followed up according to the schedule.

In this study, in addition to our conventional approach, the patients were co-treated with oral doxycycline (DoxyHEXAL® 200 mg; Hexal, Holzkirchen, Germany) once a day for 4 months after the first injection of bevacizumab. They were trained to take doxycycline correctly and were informed about the symptoms of disease activation; besides, they were screened for the incidence of pseudotumor cerebri, hepatotoxicity, azotemia, photosensitivity, rash, oral or vaginal candidiasis, and gastrointestinal adverse effects. The following data were collected: age, sex, history of cardiovascular diseases, hypertension, smoking history, type and size of lesion, initial and final visual acuity score based on Early Treatment Diabetic Retinopathy Study (ETDRS) charts, central thickness of the fovea according to the OCT reports, need for re-treatment, number of consumed doxycycline tablets, and total number of injections needed to control the disease in the first year. Active lesions of nAMD were categorized into occult, predominantly classic, and minimally classic. All patients with active nAMD were entered into the study regardless of their lesion subtype or baseline lesion size and visual acuity; however, in patients with bilateral involvement, only the left eye was included. On the condition that a patient’s best corrected visual acuity (BCVA) was below the lower threshold of standard ETDRS charts, the visual acuity score was calculated using the logarithm of the minimum angle of resolution (logMAR) equivalent value, which was obtained by conversion of counting fingers results to the logarithmic scale. In this study, severe visual loss or significant visual gain was considered when the visual acuity score changed by > 15 points.

**Table 1:** Inclusion/Exclusion Criteria and Re-treatment Indications

Inclusion criteria
Subfoveal choroidal neovascularization (CNV) due to age-related macular degeneration (AMD)
Lesion size ≤2.5 disc diameter)
Treatment-naïve patients
No history of systemic disease or myocardial infarction
Age >50 years old
Exclusion criteria
Any history of hypersensitivity to tetracyclines or a family history of breast cancer
Pregnant or lactating women
Previous treatment for neovascular AMD, subfoveal or juxtafoveal laser treatment, or a positive history of ocular surgery
CNV due to causes other than AMD
Patients with any retinal vasculopathies, including diabetic retinopathy, retinal vein occlusions, vitreomacular adhesion, fibrovascular scar, or geographic atrophy in the study eye
Acute ocular or periocular infection
Glaucoma or intraocular pressure in the study eye >22 mmHg
Re-treatment indications
Active CNV on examination or imaging
Intraretinal or subretinal fluid or leakage
New-onset macular hemorrhage
Visual acuity depletion more than 5 letters

The Shapiro–Wilk test was employed to evaluate normal distribution of quantitative variables. One-sample t-test, independent samples t-test, and paired-samples t-test were run to assess the significance of the differences between mean values. SPSS version 19.0 (SPSS Inc., Chicago, IL, USA) was used to carry out all statistical analyses. The significance level was set at P < 0.05. Data are presented here as means ± standard deviation (SD).

## RESULTS

Six patients out of the initial 28 study subjects were excluded from the study because of either lack of adhesion to the therapeutic plan or loss to follow-up. The mean age of our remaining subjects was 75.5 ± 7.7 years. Of the 22 remaining patients, 12 (54.5%) were female. Hypertension and a positive history of smoking were found in 13 (59.1%) and 8 (36.4%) patients, respectively. Eye involvement (laterality) was as follows: right in 9 cases (40.9%), left in 4 (18.2%), and bilateral in 9 (40.9%). The type of lesion was as follows: occult in 6 cases (27.3%), predominantly classic in 13 (59.1%), and minimally classic in 3 (13.6%). On the basis of the optic disc diameter (DD), the average initial lesion size was 1.25 ± 0.65 DD. Mean initial and final visual acuity scores based on ETDRS charts were 28.5 ± 22.57 and 36.45 ± 26.66, respectively. According to the OCT reports, mean initial and final central foveal thickness were 445.14 ± 63.69 m and 212.45 ± 29.50 m, respectively. Five patients (22.7%) had a significant increase in visual acuity (i.e., an increase of >15 points in the visual acuity score) and 6 (27.3%) had mild to moderate visual gain. Two patients (9%) experienced mild to moderate visual loss and 1 (4.5%) experienced a decrease in visual acuity depletion of >15 points. Visual acuity did not change in 8 patients (36.5%). Among the patients who completed the study, five complained about gastrointestinal side effects such as heartburn and dyspepsia. There was no evidence for pseudotumor cerebri, hepatotoxicity, azotemia, photosensitivity, rash, or oral or vaginal candidiasis. The data of six patients who were lost to follow-up were excluded from the statistical analysis. All quantitative variables followed a normal distribution (P > 0.05 by Shapiro–Wilk test). Sex, history of hypertension, and smoking did not affect the size or thickness of the lesion, visual acuity score, or the number of injections required for disease control (P > 0.05 for all comparisons). Co-treatment of active nAMD with intraocular bevacizumab and oral doxycycline improved the final visual acuity score and reduced the final central foveal thickness compared to initial records (mean visual acuity score: 36.45 ± 26.6 points vs. 28.5 ± 22.5 points, respectively, P = 0.03; mean foveal thickness: 212.45 ± 29.5 m vs. 445.14 ± 63.7 m, respectively, P < 0.001 by paired-sample t-test). After 12 months of follow-up, the average total number of injections needed to control disease activity was 3.18 ± 0.79 injections/year (Table 2). 

**Table 2:** Patient Baseline Characteristics and Response to Co-Treatment of nAMD by Intraocular Bevacizumab and Oral Doxycycline. Data are Means ± Standard Deviation unless Otherwise Stated

	Values
Age, years	75.5 ± 7.7
Sex, female:male ratio, n (%)	12 (54.5%):10 (45.5%)
Initial lesion size, disc diameter	1.25 ± 0.65
Initial visual acuity score *	28.5 ± 22.57
Initial thickness of the foveal central portion, m	445.14 ± 63.69
Visual acuity score change	7.92 ± 16.89 (P = 0.03)
Foveal thickness change, m	232.68 ± 69.25 (P < 0.001)
Final visual acuity score	36.45 ± 26.66
Final central foveal thickness, micrometer	212.45 ± 29.50
Number of consumed doxycycline tablets	212.5 ± 7.5
Total number of injections in 12 months	3.18 ± 0.79

* based on Early Treatment Diabetic Retinopathy Study (ETDRS) charts

## DISCUSSION

Nowadays, various anti-VEGF agents, such as pegaptanib, bevacizumab, ranibizumab, and aflibercept, are available to control nAMD lesions. The better outcomes of bevacizumab compared to ranibizumab and its affordable price compared to ranibizumab and aflibercept, make off-label application of bevacizumab the first choice in many clinical centers, including ours [6]. Due to the burden of AMD treatment on health care systems and patients, the overall tendency is to reduce the total number of intraocular injections or improve the results of standard management in more patients [1]; besides, concerns about frequent intraocular injections may limit the administration of anti-VEGF agents in patients with AMD. Although there are some doubts about the outcomes of anti-VEGF administration by treat-and-extend or as-needed regimens [6, 7], these protocols—which are based on periodic examinations, disease status, and a patient's need for re-treatment—have more acceptance than either fixed doses or monthly administration of anti-VEGF agents [8, 9]. Rather than generating new anti-VEGF agents, such as brolucizumab, designed ankyrin repeat proteins (DARPins), and topical tyrosine kinase inhibitors [10], many trials have been using anti-VEGF agents in combination with other treatments such as photodynamic therapy (PDT) [11], stereotactic radiation therapy (SRT) [12], epimacular brachytherapy, and other mediators of angiogenesis and inflammation [10, 13-15]. However, the results are controversial. According to impact of tetracyclines -especially doxycycline- on inflammation, immunomodulation, cell proliferation and angiogenesis, we decided to study the efficacy of doxycycline in reducing the total number of intraocular injections in a year. nAMD is characterized by CNV and the formation of abnormal vessels, which may leak and lead to subretinal collections. VEGF is involved in both CNV and vascular hyperpermeability from new and old vessel beds and is currently the main target for pharmacological treatment of active nAMD. Matrix metalloproteinases (MMPs) also contribute to the progression of AMD. MMPs, especially MMP-2 and MMP-9, decrease the levels of pigment epithelium-derived factor (PEDF), the main antagonist of VEGF in the retinal pigment epithelium (RPE). Furthermore, VEGF induces MMP-9 activity by increasing nitric oxide levels.

Initially, the effects of doxycycline on MMPs were considered because of its effects on angiogenesis, but further studies revealed more mechanisms beyond the inhibition of MMPs [5, 16-18]. In addition to undesirable angiogenesis in nAMD, endothelial cell junctions in new vessels are more permeable than mature ones, and VEGF aggravates this impairment. Doxycycline could prevent the effects of VEGF while inducing vascular endothelial (VE)-cadherin expression on endothelial cells [19]. In 2006, Dehrah et al. stated that doxycycline could decrease the serum level of VEGF in lymphatic filariasis [20]. Pimenta et al. showed that 12-month administration of doxycycline is safe and effective for the treatment of lymphangioleiomyomatosis and for lowering urine and plasma levels of MMPs; however, the authors concluded that the therapeutic outcomes could be not explained solely by MMP blockade [21, 22]. There is increasing evidence for the protective effect of topical or oral doxycycline against corneal neovascularization [23, 24]. In 2009, Samtani et al. successfully inhibited the progression of CNV in rats by orally administration of doxycycline [17]. Likewise, Cox et al. had similar results in a murine model of CNV and pterygium growth [25]. It has been demonstrated that co-treatment of corneal angiogenesis with doxycycline and bevacizumab is more effective than monotherapy [26]. However, in one pilot study by Sivaprasad et al., the outcomes of foursome combination therapy for nAMD with PDT, intravitreal ranibizumab, dexamethasone, and oral minocycline were considered equivalent to those of combination treatment with standard PDT and ranibizumab [27]. In 2012, Robert et al. reported the successful treatment of CNV due to *Bartonella henselae* in a young patient with doxycycline and intravitreal bevacizumab [28]. In our study, no significant side effects, such as bleeding, cardiovascular events, stroke, and sudden death, were observed. The only reported side effect was heartburn in 5 patients (22.7%), which resolved after following the instructions. Symptom severity did not interfere with the continuation of the study.

As mentioned above, it has been inferred that co-treatment of nAMD with intraocular bevacizumab (number of injections in the first year, 3.18 ± 0.79) and oral doxycycline was effective in visual acuity improvement and reduction of foveal thickness (visual acuity: 7.92 ± 16.89 points, P = 0.03; foveal thickness: 232.68 ± 69.25 m, P < 0.001). Although there were some differences between the baseline characteristics of our patients and those of comparable studies with a PRN anti-VEGF protocol, the present visual acuity changes are in agreement with previously reported outcomes (P > 0.05 in both studies) [27, 29]. Compared to the previous studies, in our patients, foveal thickness regression was greater (foveal thickness change: 66.3 m and 85.3 m versus 232.68 m, respectively, P < 0.001), but this difference could be interpreted as a consequence of the worse initial situation in our subjects. Since our study included all patients, we concluded that the combination therapy with oral doxycycline could be useful for patients with advanced disease as well as cases with early lesions. While in our case series, the total number of injections was 3.18 ± 0.79 injections/year in average (P < 0.001), in studies with similar re-treatment indications with a PRN protocol for intravitreal bevacizumab monotherapy, the mean total number of injections was 7.7 and 5.92 in the first year, respectively [6, 29]. Our results are in agreement with another pilot study that evaluated the effect of combination therapy with PDT, intravitreal ranibizumab, dexamethasone, and oral minocycline (P = 0.212) [27]. Although the authors concluded their results were similar to those of clinical trials on combination therapy with PDT and anti-VEGF, oral administration of tetracyclines, especially doxycycline, is as effective as co-treatment of CNV with standard PDT. There are numerous limitations in this study, namely the small sample size, lack of a control group, inability to evaluate doxycycline plasma levels, and single-center design. The low number of subjects did not allow us to interpret the results according to the baseline characteristics of the patients. The short follow-up time and loss to follow-up prevented us to uncover whole adverse effects. Unfortunately, we were unable to identify the cause of loss to follow-up or inappropriate adhesion to the therapeutic plan by the six excluded patients. In conclusion, even in patients with advanced nAMD, oral administration of doxycycline could reduce the need of intravitreal anti-VEGF re-injection. Larger, controlled clinical trials are needed to confirm the role of tetracyclines in the co-treatment of nAMD.
